# A New Manual of Anatomy, Descriptive and Analytical. With an Atlas of 34 Plates

**Published:** 1842-04

**Authors:** 


Art. V.—*Nouveau Manuel d'Anatomie descriptive et raisonnée, avec un Atlas de* 34 *planches noires, coloriées et decoupées.* Par C. Crommelinck, Docteur en Médecine, en Chirurgie et en l'art des Accouchemens à Bruges, &c.—*Bruxelles,* 1841. 8vo, pp. 436.

*A New Manual of Anatomy, descriptive and analytical. With an Atlas of* 34 *Plates.* By C. Crommelinck, Doctor of Medicine, Surgery, and Midwifery at Bruges, &c.—*Brussels,* 1841.

In the ordinary way of teaching, remarks M. Broc,* the necessity of gradually introducing the novice into the temple of science is never thought of. The professor being there himself supposes others there also—he does not first call attention to a general view of the whole at some distance, so as to familiarize the pupil with the larger masses, and thus prepare him to comprehend the minute details. How far M. Broc is justified in the above statement as regards teachers of anatomy, against whom it is more particularly directed, we stop not to enquire, but proceed to state that his plan consists in first giving, by means of comparison, an idea of the disposition of all the apparatuses and of the mode in which they perform their functions; he then passes to the description of the individual apparatuses and functions, but in a very general way. After this first section of his first part, he proceeds to the second section, in which he treats of the intellectual functions. Passing now to the second part, he considers the organs with reference to their principal dispositions. Lastly in the third part he gives a more minute description of the organs.

We believe M. Broc is a very popular and successful teacher—*prima facie* evidence that his plan possesses many advantages. The work of M. Crommelinck is so close a copy of that of M. Broc, both in plan and matter, that it is not necessary to say much of it. We may remark however, that in looking over it we have found great deficiencies in all that relates to the finer points of anatomy—all that in fact constitutes anatomy a science.

* Introduction a l'Etude de l'Anatomie, ou l'Homme consideré en grand sous le rapport des Appareils et des Fonctions. Paris, 1837.

